# Using the Water Absorption Ability of Dried Hydrogels to Form Hydrogel-Supported Lipid Bilayers

**DOI:** 10.3390/gels9090751

**Published:** 2023-09-15

**Authors:** Che-Lun Chin, Lu-Jan Huang, Zheng-Xian Lu, Wei-Chun Weng, Ling Chao

**Affiliations:** Department of Chemical Engineering, National Taiwan University, Taipei 10617, Taiwan

**Keywords:** supported lipid bilayers (SLBs), hydrogel, lipid vesicle deposition

## Abstract

The formation of supported lipid bilayers (SLBs) on hydrogels can act as a biocompatible anti-fouling interface. However, generating continuous and mobile SLBs on materials other than conventional glass or mica remains a significant challenge. The interaction between lipid membrane vesicles and a typical hydrogel is usually insufficient to induce membrane vesicle rupture and form a planar lipid membrane. In this study, we demonstrate that the water absorption ability of a dried polyacrylamide (PAAm) hydrogel could serve as a driving force to facilitate the formation of the hydrogel–SLBs. The absorption driving force vanishes after the hydrogels are fully hydrated, leaving no extra interaction hindering lipid lateral mobility in the formed SLBs. Our fluorescence recovery after photobleaching (FRAP) results show that SLBs only form on hydrogels with adequate absorption abilities. Moreover, we discovered that exposure to oxygen during drying could lead to the formation of an oxidized crust on the PAAm hydrogel surface, impeding SLB formation. Therefore, minimizing oxygen exposure during drying is crucial to achieving high-quality hydrogel surfaces for SLB formation. This water absorption method enables the straightforward fabrication of hydrogel–SLBs without the need for additional substrates or charges, thereby expanding their potential applications.

## 1. Introduction

Supported lipid bilayers (SLBs) are a popular cell membrane-mimicking model which could be applied in coatings or used as interfaces to investigate membrane-related phenomena [1]. Given that some of the head groups of lipid bilayers are hydrophilic and zwitterionic, and could strongly bind water molecules to form a hydration layer [2], the SLBs possess excellent anti-fouling properties. The property can drastically decrease the nonspecific adsorption of biomolecules and the adhesion of cells and bacteria to the surface. Therefore, the lipid bilayer has been used as a coating for anti-fouling applications in protein-rich environments, such as in coatings of microgels for drug delivery [3], coatings to improve the problem of fouling [4,5] and clogging [6] in microchannels, and coatings to reduce nonspecific adsorption in immunoassays [7,8].

The antifouling characteristic made SLBs vital in a variety of applications. Most studies still construct SLBs on conventional solid materials like fused silica, borosilicate glass, and mica [9,10]. However, the mechanical properties of solid materials make bending difficult, limiting the possibility of bio-applications. In comparison, hydrogels could be satisfying soft substrates for SLBs. Their biocompatibility has been shown in commercialized products, including contact lenses, tissue engineering scaffolds, and wound dressings [11]. The tunable mechanical strength of hydrogels also makes it easier to adjust for various applications [12].

However, it is still difficult to deposit lipid vesicles to form SLBs on substrates other than mica and glass. Previous studies have employed techniques such as atomic force microscopy (AFM) [13,14] and quartz crystal microbalance (QCM) [15,16] to demonstrate that SLB formation via vesicle fusion involves the adsorption of vesicles on the substrate, followed by their deformation and rupture. Upon rupture, the lipid vesicles can fuse and reorganize, resulting in the formation of a continuous SLB on the substrate [17,18,19]. Crucial to this process is a sufficient attractive force between the vesicles and the substrate, which induces vesicle deformation [20,21,22]. Such deformation leads to the occurrence of some high curvature regions in the lipid membrane, significantly increasing the likelihood of vesicle rupture [23,24]. To date, few methods have been reported to construct SLBs on hydrogels, likely due to the weak interaction between the hydrogels and lipid membrane vesicles.

There have been several attempts to build SLBs on soft materials such as polymers or hydrogels. Some studies have increased the electrostatic attraction between the lipid membrane and soft substrates to induce vesicle rupture [25,26,27]. Although the electrostatic attraction could facilitate the rupture of lipid vesicles, nevertheless, it also hinders the lateral mobility of lipids after SLB formation, which could restrict further applications. The other studies applied thin, soft material cushions on glass and used the attraction from glass to induce the SLB formation [28]. However, the thickness of the soft material cushion is limited because lipid vesicles rely on the attraction of glass to deform and rupture and cannot be far away from the glass substrate.

Herein, we propose that the water absorption ability of hydrogels could act as an additional and temporary driving force to induce the formation of SLBs on the hydrogels. The water absorption ability of a dried gel could compress and rupture lipid vesicles to form SLBs during swelling. The absorption driving force would vanish after the hydrogels are fully hydrated, leaving no additional attraction to hinder the lateral mobility of the SLBs. In addition, since the driving force does not come from the glass support below the hydrogel, the hydrogel thickness is not limited as long as the hydrogel can provide sufficient absorption ability. In this study, we used polyacrylamide (PAAm) hydrogel to conduct our experiment because of its ease of fabrication, electroneutrality, and biocompatibility. To verify the proposed mechanism, we deposited lipid vesicles on PAAm hydrogels with various absorption abilities and used fluorescence recovery after photobleaching (FRAP) to examine the integrity of the formed SLBs.

## 2. Results and Discussion

### 2.1. Adjusting Water Absorption Ability to Induce Vesicle Rupture

**Figure 1:**
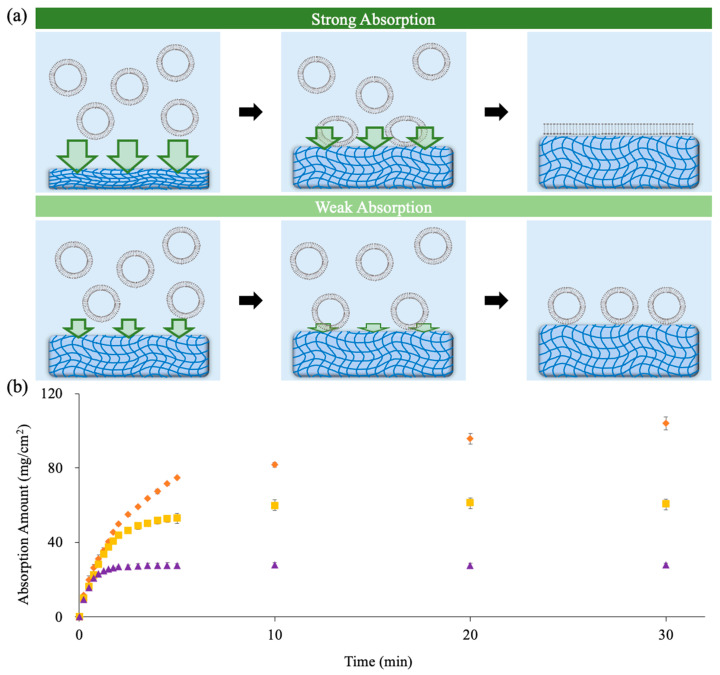
(**a**) A schematic illustration of the proposed situation when lipid vesicles are added to hydrogels with strong and weak absorption abilities during the swelling process. When lipid vesicles are added to a hydrogel with strong absorption ability, they are brought down onto the hydrogel surface, compressed, and ruptured to form SLBs. On the other hand, when lipid vesicles are added to a hydrogel with weak absorption ability, they cannot rupture to form SLBs. (**b**) Buffer absorption profiles of PAAm hydrogels with different thicknesses (235 μm in orange, 120 μm in yellow, and 60 μm in violet). The values were obtained by averaging three sets of data.

Figure 1a illustrates our hypothesis that the water absorption ability of a dried hydrogel could be used as a driving force to induce SLB formation. After the addition of lipid vesicles to a dried hydrogel, the water absorption could first induce a solution flow to drive the vesicles to the hydrogel surface. If the water absorption ability is strong enough, the vesicles on the hydrogel surface could be compressed. With sufficient compression, the edges with high curvature could become rupture sites for the vesicle to rupture, and the SLB will then form. On the other hand, if the water absorption ability is insufficient to compress vesicles, the vesicles would be washed away or remain adsorbed on the hydrogel surface.

To verify our proposed hypothesis, we first prepared PAAm hydrogels with various thicknesses and initial water contents (IWCs) to adjust the hydrogel water absorption ability. Figure 1b shows the absorption amount profiles of PAAm hydrogels with thicknesses of 235 ± 7 μm, 120 ± 1 μm, and 60 ± 3 μm. The absorption reached equilibrium about 30 min after hydrogels were soaked in our buffer solution. The equilibrium buffer uptake levels of 235 μm, 120 μm, and 60 μm PAAm hydrogels were 109 ± 3 mg/cm^2^, 60 ± 3 mg/cm^2^, and 28 ± 2 mg/cm^2^, respectively. The water absorption ability of a hydrogel refers to the absorption flux (absorption amount per unit area per unit time) and can be obtained from the slopes of the absorption profiles in Figure 1b. The slopes in Figure 1b indicate that the fluxes of the hydrogel, with three varying thicknesses, were similar during the initial stages. However, the thinner hydrogel reached the saturation state more rapidly, resulting in a swift decline in flux.

### 2.2. Formation of SLBs on Polyacrylamide Hydrogel with Various Absorption Abilities

To prove that sufficient absorption ability is required to induce the SLB formation, we deposited lipid vesicles on PAAm hydrogels with various absorption abilities. We used the fluorescence recovery after photobleaching (FRAP) method to examine whether an SLB formed. To achieve this, a small fraction of a fluorescent lipid probe, Texas-Red DHPE, was incorporated into the lipid membrane vesicles. If the vesicles ruptured to form a continuous SLB, the fluorescently labeled lipids could move laterally and freely within the planar membrane. During the FRAP experiment, a laser was used to selectively bleach a region of the fluorescent probes in the SLB. As a result, the probes in neighboring regions could diffuse and exchange with the bleached probes laterally, leading to the recovery of fluorescence intensity in the bleached region over time. By analyzing the fluorescence-intensity-recovery profiles over time, we can obtain the diffusivities and mobile fractions of the formed SLBs. The magnitude of diffusivities reflects the state of the lipid membrane, and a mobile fraction close to one indicates the presence of a continuous membrane with minimal defects or holes.

**Figure 2:**
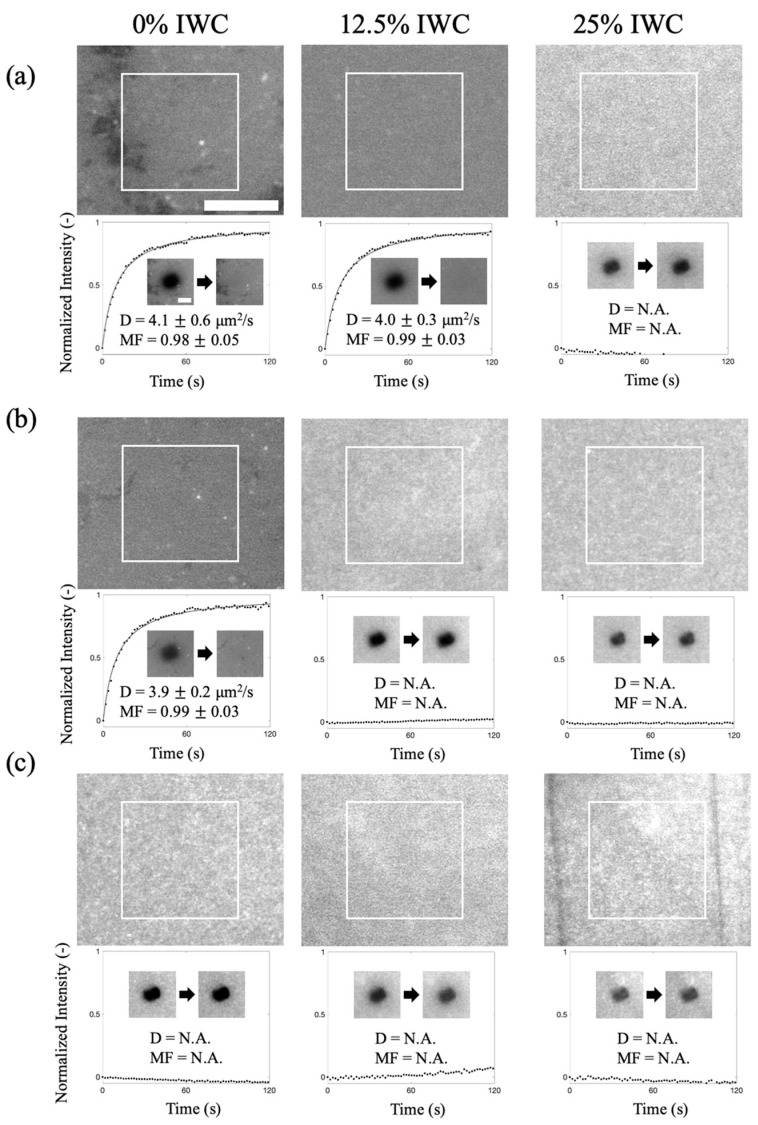
Fluorescence images and FRAP recovery profiles of lipid vesicle deposition on PAAm hydrogels with three different thicknesses: (**a**) 235 μm, (**b**) 120 μm, and (**c**) 60 μm, respectively. Diffusivity (D) and mobile fraction (MF) provide integrity information about the deposited lipid membranes. The values were obtained by averaging five sets of data. The scale bar was 50 μm for the large images and 25 μm for the smaller images with the recovery profiles.

Figure 2 shows the fluorescence recovery results of the samples after lipid membrane vesicles were added to the dried hydrogels with various absorption abilities. We prepared hydrogels with thicknesses of 235 μm and 120 μm, along with varying levels of IWC (0%, 12.5%, and 25%). The corresponding absorption abilities are experimentally measured and listed in Table 1. We found that the fluorescence intensities in the bleached spots successfully recovered on the 235 μm hydrogel, with 0% IWC, and on the 235 μm hydrogel with 12.5% IWC (Figure 2a). The measured diffusivities (4.1 ± 0.6 μm^2^/s and 4.0 ± 0.3 μm^2^/s) were comparable to those measured in SLBs on glass [29,30], and the mobile fractions were close to one, suggesting the formation of continuous SLBs on the hydrogels. On the other hand, the bleached spots were not recovered on the 235 μm hydrogel with 25% IWC. The observed fluorescence intensity of the sample may be attributed to the presence of unruptured vesicles adsorbed on the surface. Since the fluorescence probes in one vesicle could not easily exchange with those in the other vesicles, the fluorescence of bleached vesicles was unable to recover. This result suggests that the absorption ability of the 235 μm hydrogel with 25% IWC (24 mg/cm^2^·min) was already too weak for the vesicles to rupture to form continuous SLBs.

Figure 2b,c shows the fluorescence images and FRAP results of the 120 μm and 60 μm thick hydrogels after lipid vesicle deposition. The FRAP results indicate that SLBs also formed on the 120 μm hydrogel with 0% IWC, but not on the 60 μm hydrogel with 0% IWC. Although the initial flux of the 60 μm hydrogel with 0% IWC (44 mg/cm^2^·min) was similar to that of the 120 μm (43 mg/cm^2^·min) hydrogels, the flux dropped quickly with time and probably could not sustain sufficient fluxes long enough for the vesicles to rupture. This phenomenon is consistent with previous studies, indicating that it takes some time for the vesicles to rupture and reorganize, leading to the formation of SLBs [20,22,31]. In addition, since the 120 μm hydrogel with 12.5% IWC (36 mg/cm^2^·min), the 60 μm hydrogel with 12.5% IWC (39 mg/cm^2^·min), and the 60 μm hydrogel with 25% IWC (34 mg/cm^2^·min) had even smaller fluxes than the 60 μm hydrogel with 0% IWC (44 mg/cm^2^·min), it is reasonable that there was also no fluorescence recovery on these hydrogel surfaces.

To further quantify the flux decay, we defined a duration based on the time taken for the flux to decrease to the initial flux of the 235 μm hydrogel with 25% IWC (24 mg/cm^2^·min). Table 1 shows that the absorption durations are shorter in the thinner hydrogels, explaining why no continuous SLB formed on the 60 μm PAAm with 0% IWC. Without sufficient absorption time, even with an adequate initial absorption flux, the SLB cannot form.

### 2.3. Exposure to Oxygen during Shrinkage Hinders SLB Formation on the Hydrogel Surface

**Figure 3:**
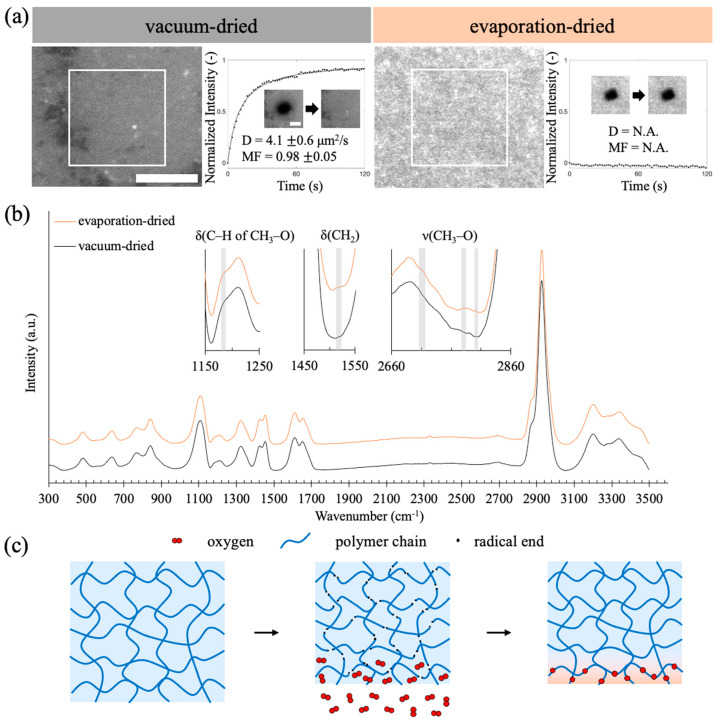
(**a**) Fluorescence images and FRAP recovery profiles of lipid vesicle deposition on the 235 μm PAAm hydrogels fabricated with vacuum-dried and evaporation-dried processes, respectively. The diffusivities and mobile fractions were obtained by averaging three sets of data. (**b**) Raman spectra of the evaporation-dried and vacuum-dried hydrogels. The Raman spectra of vacuum-dried PAAm are labeled in black, while the Raman spectra of evaporation-dried PAAm are labeled in orange. The shaded regions indicate the peaks exclusively observed in the evaporation-dried PAAm hydrogel. (**c**) A schematic illustration of the oxidized layer formation. The shrinkage of PAAm hydrogel during drying could cause mechanical stress to induce radical formation (black dots). An oxidized layer could occur at the PAAm hydrogel surface, where the radicals can be exposed to oxygen.

We found that the handling of the drying process in PAAm hydrogel fabrication affects the outcome of lipid vesicle deposition on PAAm hydrogels. After the polymerization process (a free radical chain reaction) of PAAm hydrogel was finished, it had to be dried before lipid vesicle deposition to provide sufficient absorption ability. The FRAP results in Figure 3a show that SLBs can form on a vacuum-dried PAAm hydrogel surface, but not on an evaporation-dried PAAm hydrogel surface with exposure to the air during the drying. We also found that a vacuum-dried hydrogel surface exhibits a higher degree of hydrophilicity in comparison to an evaporation-dried hydrogel surface (Figure S1). Moreover, SLBs could still form on the inner surface cut of an evaporation-dried hydrogel (Figure S2). We therefore hypothesized that some reactions associated with oxygen exposure might occur at the surface of the hydrogel.

We employed Raman spectroscopy to investigate potential alterations in composition resulting from reactions associated with exposure to oxygen on the surface of evaporation-dried hydrogels. It is postulated that alkyl radicals present in PAAm may engage in reactions with oxygen, leading to the formation of peroxy radicals [32,33]. These peroxy radicals, in turn, have the capacity to abstract hydrogen atoms from alkyl chains or react with alkyl radicals, resulting in the generation of R-O-O-H and R-O-O-R (dialkyl peroxide) species [34]. Considering the relatively weak bond energy of O-O, it is plausible for this bond to be disrupted by light under normal conditions, thereby yielding alkoxy radicals [35,36]. Alkoxy radicals can subsequently participate in reactions with alkyl or peroxy radicals, culminating in the formation of ether bonds [32,34,37]. We examined whether Raman peaks associated with these oxidized products appeared in the Raman spectrum of evaporation-dried hydrogels.

In Figure 3b, the Raman spectra of both vacuum-dried and evaporation-dried PAAm hydrogels are presented. The spectra are similar and both spectra exhibit similarities to those documented in the literature [38,39,40]. Given our hypothesis, that oxidation primarily occurs at the surface, it was anticipated that the peaks corresponding to the oxidized products might exhibit relatively low intensity. We magnified the spectra and discerned the presence of several peaks in the evaporation-dried hydrogels, which were absent in the PAAm spectra previously reported in the literature. The shaded regions in Figure 3b highlight five distinct peaks at 1185 cm^−1^, 1516 cm^−1^, 2713 cm^−1^, 2778 cm^−1^, and 2804 cm^−1^ observed in the evaporation-dried hydrogels. These peaks could be attributed to the bonds in ethers (deformation of C–H in CH_3_–O at 1185 cm^−1^, deformation of CH_2_ at 1516 cm^−1^, and stretching of CH_3_–O at 2713 cm^−1^, 2778 cm^−1^, and 2804 cm^−1^), and cannot be found in pure PAAm [41,42,43,44,45]. The emergence of these additional peaks provides compelling evidence in support of the occurrence of surface oxidation.

We washed the hydrogels after the polymerization step to remove residue initiators, and it remains unclear what can reinitiate the potential oxidation reaction or the formation of alkyl radicals in the PAAm matrix during the subsequent drying process. Previous studies have suggested that mechanical stress can induce the homolytic cleavage of the polymer C-C backbones, generating alkyl radicals at the scission ends [32,46,47]. During the drying process, PAAm hydrogels undergo shrinkage, which can be considered a form of mechanical stress. When newly formed alkyl radicals come into contact with oxygen, the reaction mentioned above can occur, leading to the formation of an oxidized surface layer with ether groups.

### 2.4. Severe Expansion in the Horizontal Plane of the Evaporation-Dried Hydrogel Produces a Crack Structure

In our standard procedure, we applied weight on top of the hydrogel during the drying process. This ensured minimal changes in the horizontal dimensions of the hydrogel and prevented the excessive expansion of the hydrogel’s lateral area during water absorption, which could potentially lead to the disruption of the formed SLB above the hydrogel.

**Figure 4:**
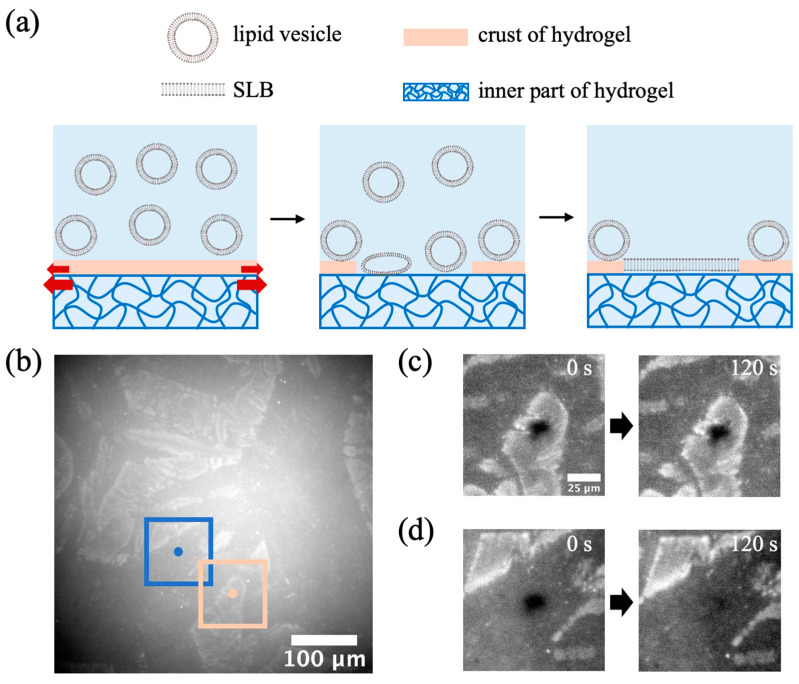
(**a**) A schematic illustration of the swelling process of the hydrogel surface with severe expansion in the horizontal plane. When the hydrogel starts to swell, the crust splits, and the inner part of the hydrogel becomes exposed to the lipid vesicle solution. The lipid vesicles ruptured to form SLBs on the inner part of the hydrogel, but not on the crust. The red arrows indicate that the different swelling extents of the oxidized surface layer and the inner part of hydrogel may cause the crack of the surface layer. (**b**) Fluorescence image of lipid vesicle deposition on a 235 μm thick PAAm hydrogel fabricated using the evaporation-dried process without applying any weight during the drying stage. FRAP recovery profiles of lipid vesicle deposition on the (**c**) crust and (**d**) inner part, respectively. The FRAP images in (**c**,**d**) were obtained from locations marked by blue and pink boxes, respectively, in (**b**). The dots at the center of each box indicate the positions where the lasers were applied during the experiment.

To further demonstrate that the oxidation process we discussed earlier only occurs at the surface of the hydrogel, we prepared an evaporation-dried PAAm hydrogel without placing any weight on top during the drying process. The resulting lateral area of the hydrogel decreased to 49% of its original size after drying and expanded to 172% of the dried hydrogel’s dimensions upon full hydration (Figure S3). As discussed in the previous subsection, the shrinkage process of the evaporation-dried hydrogel could lead to the formation of an oxidized layer. It is important to note that this oxidized layer may have a smaller swelling extent compared to the inner part of the hydrogel. Consequently, cracks can develop on the surface layer during the hydration process, potentially exposing parts of the inner hydrogel. The exposure of lipid vesicles to different surfaces can give rise to varied deposition patterns, as depicted in Figure 4a.

The fluorescence image in Figure 4b reveals a crack-like structure after the deposition of lipid vesicles. Figure 4c displays the FRAP results obtained from lipid membranes on the crust surface and the inner part of the hydrogel. The fluorescence recovery was observed on the surface of the inner part of the hydrogel, while no recovery was observed on the crust surface. This outcome supports the previously mentioned mechanism of oxidized crust formation, indicating that the oxidized outer layer is relatively thin compared to the overall thickness, rendering it unsuitable for SLB formation.

## 3. Conclusions

Our findings demonstrate that SLBs can form on PAAm hydrogels with a sufficient water absorption capacity. This suggests that the ability of the hydrogel to absorb water can serve as a driving force for inducing SLB formation. When it comes to fabricating dried hydrogels, we discovered that it is crucial to maintain an oxygen-deprived environment during the drying process to prevent oxidation. Our Raman spectra analysis suggests that the exposure of PAAm hydrogel to oxygen in the air during shrinkage may trigger an oxidation reaction, resulting in the formation of an oxidized surface crust. This crust could impede SLB formation, as evidenced by the FRAP results. Some previous studies have increased electrostatic attraction between lipid vesicles and soft substrates for hydrogel–SLB formation. However, this attraction limits lipid mobility after the SLB formation. Other studies used thin hydrogel cushions on silica substrates, but thickness is limited due to the reliance on glass attraction for vesicle deformation and rupture. In this study, the absorption-driven force diminishes once the hydrogel is fully hydrated, thereby eliminating any additional factors that could hinder the lateral mobility of the SLBs. The hydrogel–SLB interaction is independent of the underlying solid support, allowing for greater flexibility in hydrogel thickness. This water absorption method enables the straightforward fabrication of hydrogel–SLBs without additional solid supports or charges, expanding their potential applications.

## 4. Materials and Methods

### 4.1. Materials

The 30% acrylamide/bis-acrylamide (with an acrylamide/bis-acrylamide ratio of 37.5:1) was purchased from Bio-Rad (Hercules, CA, USA). Ammonium persulfate, *N*,*N*,*N*′,*N*′-tetramethyl ethylenediamine (TEMED), tris(hydroxymethyl)aminomethane hydrochloride (Tris-Cl), and potassium chloride (KCl) were purchased from Sigma-Aldrich (St. Louis, MO, USA). 1,2-dioleoyl-*sn*-glycero-3-phosphocholine (DOPC) was purchased from Avanti (Alabaster, AL, USA). 1,2-dihexadecanoyl-*sn*-glycero-3-phosphoethanolamine triethylammonium salt (Texas-Red DHPE) was purchased from Thermo Fisher Scientific (Waltham, MA, USA). All chemicals were of analytical grade and used without further purification.

### 4.2. Preparation of Dried PAAm Hydrogels

The solution consisted of 30% acrylamide (with an acrylamide/bis-acrylamide ratio of 37.5:1), 0.01% *w*/*v* ammonium persulfate, and 0.075% *v*/*v* TEMED which was prepared and mixed in a polystyrene conical centrifuge tube. For each experiment, a specified amount of the prepared solution was added onto a clean glass coverslip (2.4 × 3.0 cm^2^) according to the quantities outlined in Table 2. The added solution was allowed to spread across the entire coverslip, while being confined to the coverslip region due to surface tension. Subsequently, a second clean glass coverslip was gently placed on the spread solution, effectively creating a sandwich structure. The various amounts of solution mentioned in Table 1 could have uniform confinement in the gap between the two coverslips, resulting in the preparation of PAAm hydrogels with three different thicknesses. The wet hydrogel thicknesses were later measured, and the results are also provided in Table 2. To prepare vacuum-dried hydrogels, the wet gels were pressed with iron weights (1.1 × 10^4^ Pa) and subjected to a vacuum environment for 2.5 h. For the evaporation-dried PAAm hydrogels, the wet gels were also pressed with iron weights (1.1 × 10^4^ Pa) but were instead left exposed to the atmosphere for 96 h.

PAAm hydrogels with different initial water contents (IWCs) were made by adding different weights of buffer solution to the fully dried hydrogels. The dried hydrogels were considered to be fully dried if the weight loss after an additional 30 min of vacuum treatment was less than 2% *w*/*w*. The *IWC* is calculated by the ratio of the added buffer weight to the buffer absorption weight at the saturation state.

$$IWC=\frac{W}{W_{sat}}\times 100\%$$

where

*W*: weight of the added solution per unit area (mg/cm^2^);

*W_sat_*: weight of the absorbed solution at the saturation state per unit area (mg/cm^2^).

### 4.3. Calculation of the Absorption Flux of Dried Hydrogels

The calculation of the absorption flux and duration of dried hydrogels was carried out using the following process. The values of absorption flux were first calculated using three-point differential formulas with absorption amount data points. To reduce fluctuations, the flux values were then fitted using an exponential function with least square regression. The exponential functions of absorption flux were then used to calculate fluxes and durations of dried hydrogels.

### 4.4. Formation of Supported Lipid Membranes on PAAm

Briefly, 99.5 mol% DOPC and 0.5 mol% Texas-Red DHPE, mixed in chloroform, were subjected to vacuum treatment to eliminate the solvent. Subsequently, the vial containing the dried lipid film was reconstituted in Tris buffer (10 mM Tris-Cl, 150 mM KCl, pH 7.6) to achieve a concentration of 2 mg/mL. Large unilamellar vesicles (LUVs) were generated using the extrusion method, passing the lipid suspension through a 50 nm polycarbonate filter 21 times. The dried PAAm hydrogel pieces, as described in Section 4.2, were soaked in 0.5 mL of the LUV suspension for 30 min. Subsequently, 10 mL of Tris buffer was added to the container containing the soaked hydrogel pieces, effectively replacing any remaining LUV solution with the buffer.

### 4.5. Fluorescence Microscopy and FRAP

Fluorescence and bright-field images were captured using an inverted microscope, IX83, from Olympus (Tokyo, Japan), equipped with a CCD camera, ORCA-R2, from Hamamatsu (Shizuoka, Japan), and an illumination system, pE-300, from CoolLED (Andover, UK) connected to the microscope. A DPSS laser diode module with a wavelength of 532 nm from Unice (Taoyuan, Taiwan) was used to bleach Texas-Red DHPE fluorescence dye. Images were processed using ImageJ by National Institutes of Health (NIH). The FRAP images were analyzed with MATLAB R2020a software by MathWorks (Natick, MA, USA) using an algorithm we developed previously [48].

### 4.6. Raman Measurement

The hydrogel samples were measured using an inVia™ confocal Raman microscope from Renishaw (Gloucestershire, UK) with a 633 nm helium-neon laser. The laser power was controlled at 7.1 mW, and each measurement was conducted with three accumulations. Raman spectra were evaluated using WiRE 5.0 software by Renishaw (Gloucestershire, UK). All spectra were filtered using a Savitzky–Golay polynomial with an order of three and frame length of 31, background subtracted with a polynomial with an order of five, and normalized via total scanning areas from 100 cm^−1^ to 3600 cm^−1^, followed by smoothing using a moving mean with a window of 17.

## Figures and Tables

**Table 1:** Absorption ability of PAAm hydrogels with different thicknesses and initial water contents (IWCs). The duration refers to the time the absorption flux decays to 24 mg/cm^2^·min. The values were obtained by averaging three sets of data.

Thickness (µm)	IWC (-)	0%	12.5%	25%
235	initial flux (mg/cm^2^·min)	58 ± 6	41 ± 3	24 ± 5
	duration (min)	0.72 ± 0.15	0.44 ± 0.14	0.01 ± 0.19
120	initial flux (mg/cm^2^·min)	43 ± 2	36 ± 1	30 ± 1
	duration (min)	0.66 ± 0.05	0.47 ± 0.05	0.24 ± 0.06
60	initial flux (mg/cm^2^·min)	44 ± 1	39 ± 1	34 ± 1
	duration (min)	0.45 ± 0.01	0.36 ± 0.01	0.27 ± 0.01

**Table 2:** Volumes of the used solution and the corresponding hydrogel thicknesses.

**Solution volume (mL)**	0.135	0.270	0.540
**Thickness (µm)**	60 ± 3	120 ± 1	235 ± 7

## Data Availability

The data presented in this study are available on request from the corresponding author.

## Supplementary Materials

The following supporting information can be downloaded at: https://www.mdpi.com/article/10.3390/gels9090751/s1, Figure S1: Sessile drop measurement to show the hydrophilicity of the vacuum-dried and evaporation-dried PAAm hydrogel surfaces; Figure S2: Formation of SLBs on the dry-cut face of evaporation-dried PAAm hydrogels; Figure S3: Impact of weight application during the drying process on the hydrogel shrinkage ratio.
