# Supplementary material for: Using the Water Absorption Ability of Dried Hydrogels to Form Hydrogel-Supported Lipid Bilayers
Source: Gels. 2023 Sep 15;9(9):751. doi: 10.3390/gels9090751 (PMC10530932; doi:10.3390/gels9090751)
Supplement: Supplementary file 1 [file gels-09-00751-s001.zip › gels-2597850-supplementary.pdf]

Supplementary Information

Using the Water Absorption Ability of Dried Hydrogels to Form Hydrogel-Supported Lipid Bilayers

Che-Lun Chin, Lu-Jan Huang, Zheng-Xian Lu, Wei-Chun Weng, and Ling Chao
Department of Chemical Engineering, National Taiwan University, Taipei, Taiwan

Sessile drop measurement to show the hydrophilicity of the vacuum-dried and evaporation-dried PAAm hydrogel surfaces.

To assess the hydrophilicity of the vacuum-dried and evaporation-dried PAAm hydrogel surfaces, we conducted a sessile drop measurement. In this experiment, we deposited 15 μL of deionized water onto the surfaces using a pipette, and images were captured using a Canon EOS 800D camera. The measurement was performed at a temperature of 25℃ and a relative humidity of 65%.

The contact angle measurements obtained were 24° ± 1° for the vacuum-dried PAAm hydrogel and 35° ± 2° for the evaporation-dried PAAm hydrogel. These results indicate that the surface of the vacuum-dried PAAm hydrogel exhibits greater hydrophilicity compared to the evaporation-dried PAAm hydrogel. A smaller contact angle signifies a higher affinity of the surface towards the water, indicating increased hydrophilicity. This sessile drop measurement provides quantitative evidence supporting the observation that the vacuum-dried PAAm hydrogel surface has enhanced hydrophilic properties compared to the evaporation-dried hydrogel surface.

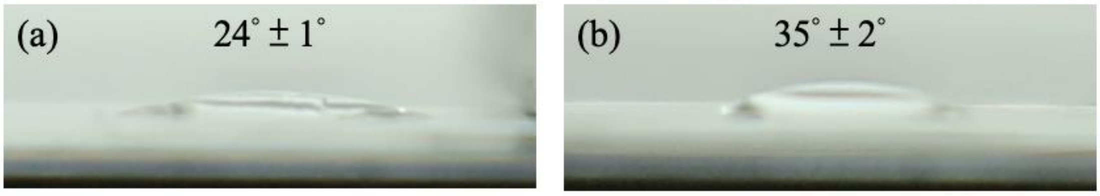

Figure S1. The images of sessile drop measurement on surfaces of (a) vacuum-dried and (b) evaporation-dried PAAm hydrogels, respectively. The values were obtained by averaging five measurements.

Formation of SLBs on the dry-cut face of evaporation-dried PAAm hydrogel.

In order to provide further evidence demonstrating the impact of oxygen exposure on the suitability of polyacrylamide hydrogel's outer surface for vesicle deposition and subsequent SLB formation, we conducted experiments depositing lipid vesicles on the cut surfaces of two different types of polyacrylamide hydrogels. Specifically, we examined the dry-cut surface and the wet-cut surface. The dry-cut surface was prepared by cutting a dried PAAm hydrogel using a blade after the evaporation process. On the other hand, the wet-cut surface was obtained by cutting a PAAm hydrogel immediately after gelation and subsequently allowing it to dry by evaporation at room temperature. Both types of hydrogel samples were exposed to air for 96 hours during the drying process. The key distinction between the two is that the surface of the dry-cut hydrogel remained within the gel during the drying process, while the surface of the wet-cut hydrogel was directly exposed to air during drying.

Figure S2 illustrates the results of fluorescence recovery after photobleaching (FRAP) following the deposition of lipid vesicles on the two different types of surfaces. Notably, the photobleached area on the dry-cut surface displayed rapid recovery, indicating the successful formation of a continuous SLB. The scratch-like defects observed on the dry-cut surface were a consequence of the cutting process. Conversely, no fluorescence recovery was observed on the wet-cut surface. These deposition results further support the notion that the oxidized crust impedes the formation of SLBs.

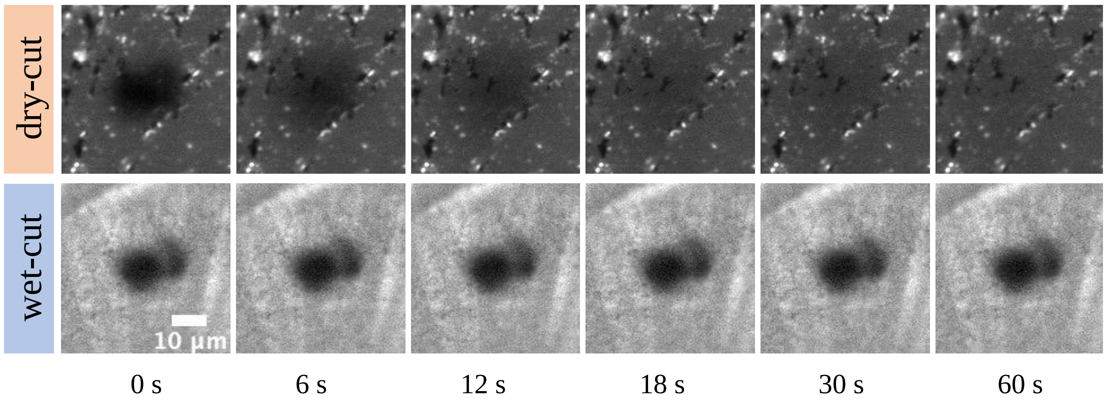

Figure S2. The fluorescence recovery after photobleaching results after the lipid vesicle deposition on the two types of polyacrylamide hydrogels: (top) dry-cut; (bottom) wet-cut.

Impact of weight application during the drying process on hydrogel shrinkage ratio.

We have discovered that the application of weight during the drying process has a significant effect on the horizontal shrinkage ratio of hydrogels. Figure S3 illustrates the changes in the horizontal area for hydrogels subjected to drying with and without the application of weight. For the hydrogel dried with weight application (1.1×10^4 Pa), the horizontal area shrank to 81.9% of the initial area after gelation. Conversely, the hydrogel dried without weight experienced a more substantial shrinkage, with the horizontal area decreasing to 49.0% of the initial area. It is worth noting that such excessive shrinkage could lead to a considerable expansion ratio during the subsequent swelling process.

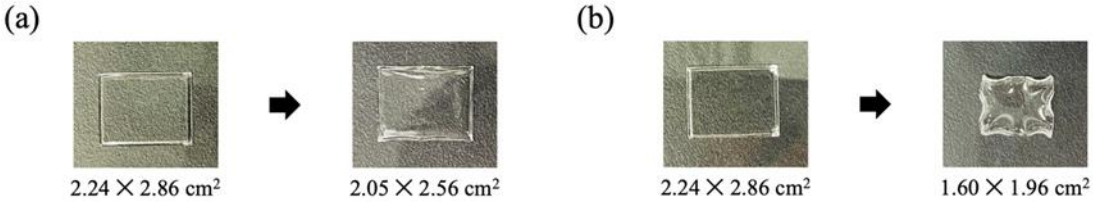

Figure S3. The horizontal surface area changes of the hydrogels dried (a) with weight application (1.1×10^4 Pa) and (b) under no weight. In each condition, the left image shows the surface area right after gelation, and the right image shows the area after drying.
